# Bite Forces and Their Measurement in Dogs and Cats

**DOI:** 10.3389/fvets.2018.00076

**Published:** 2018-04-13

**Authors:** Se Eun Kim, Boaz Arzi, Tanya C. Garcia, Frank J. M. Verstraete

**Affiliations:** ^1^Biomaterial R&BD Center, Chonnam National University, Gwangju, South Korea; ^2^Department of Surgical and Radiological Sciences, School of Veterinary Medicine, University of California, Davis, Davis, CA, United States; ^3^Department of Anatomy, Physiology and Cell Biology, School of Veterinary Medicine, University of California, Davis, Davis, CA, United States

**Keywords:** bite force, mastication, muscles of mastication, dogs, cats

## Abstract

Bite force is generated by the interaction of the masticatory muscles, the mandibles and maxillae, the temporomandibular joints (TMJs), and the teeth. Several methods to measure bite forces in dogs and cats have been described. Direct *in vivo* measurement of a bite in dogs has been done; however, bite forces were highly variable due to animal volition, situation, or specific measurement technique. Bite force has been measured *in vivo* from anesthetized dogs by electrical stimulation of jaw adductor muscles, but this may not be reflective of volitional bite force during natural activity. *In vitro* bite forces have been estimated by calculation of the force produced using mechanical equations representing the jaw adductor muscles and of the mandible and skull structure Bite force can be estimated *in silico* using finite element analysis (FEA) of the computed model of the anatomical structures. FEA can estimate bite force in extinct species; however, estimates may be lower than the measurements in live animals and would have to be validated specifically in domestic dogs and cats to be reliable. The main factors affecting the bite forces in dogs and cats are body weight and the skull’s morphology and size. Other factors such as oral pain, TMJ disorders, masticatory muscle atrophy, and malocclusion may also affect bite force. Knowledge of bite forces in dogs and cats is essential for various clinical and research fields such as the development of implants, materials, and surgical techniques as well as for forensic medicine. This paper is a summary of current knowledge of bite forces in dogs and cats, including the effect of measurement methods and of other factors.

## Introduction

Bite force is one of the significant indicators of the functional state of the masticatory system and is generated by the craniomandibular structures, including the jaw adductor muscles, temporomandibular joints (TMJs), and the teeth (1).

**Figure 1:**
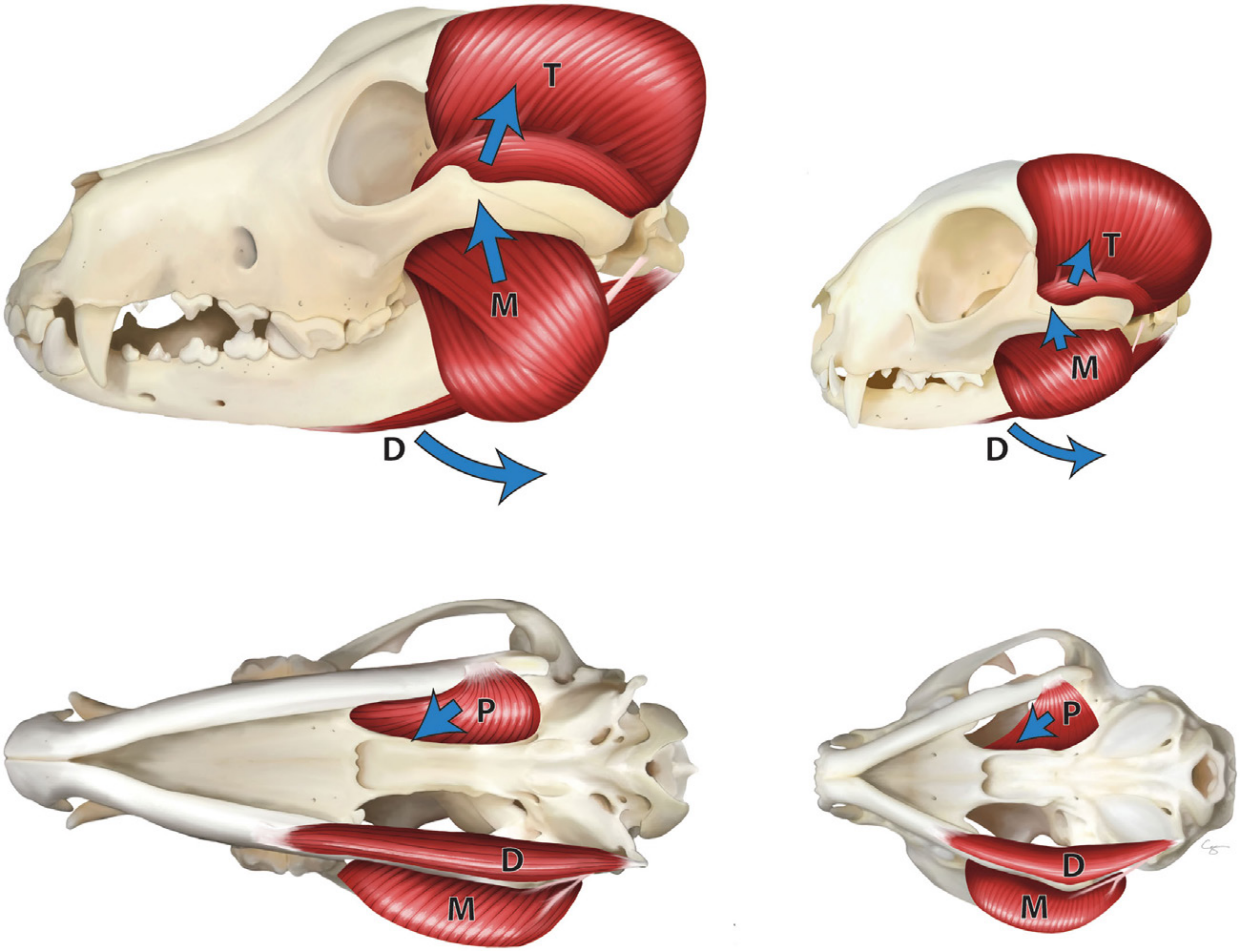
Masticatory muscles in the dog (left) and cat (right)—lateral view (top), ventral view (bottom). M, masseter muscle; T, temporal muscle; P, pterygoid muscle; and D, digastric muscles. Arrows indicate the direction of pull of the muscles.

The jaw adductor muscles play the main role in the generation of bite force in dogs and cats. These muscles include the temporal, masseter, and medial and lateral pterygoid muscles (2–4). These are the muscles that close the mouth, determine the jaw movement, and control the bite force (5) (Figure 1).

The TMJ is a synovial condylar joint formed between the head of the mandible and the mandibular fossa of the squamous part of the temporal bone (6, 7). In dogs and cats, the premolar and molar teeth have a scissor (secodont) action; thus, the mandibles are mainly moved by a hinge-like vertical motion, and lateral motion is little and limited (4, 8). Therefore, the TMJ bony components have a transversely elongated mandibular head at the mandibular side and a narrow tubular component of the mandibular fossa at the temporal bone side (4). The TMJ is crucial for masticatory function, along with dental occlusion, masticatory muscles, movement at the mandibular symphysis, and shape of the skull (9–12).

The mandibles move about the skull, and the TMJs guide it through contraction of the masticatory muscles; the force is then transmitted to the maxillary and mandibular teeth, generating the bite force (1, 13).

In humans, the shape of the skull does not differ greatly, and it is relatively easy to apply detachable sensors to teeth. However, even in humans, measuring the bite force is more challenging than measuring forces in other body regions, such as the shoulder, arm, and hip. This is because the masticatory system involves a large number of muscles of various shapes and sizes and complex architecture. Moreover, it is difficult to apply the concepts used to measure forces in other joints to the TMJ (14–16). However, there are significant differences across and even within species. In particular, in dogs, there are considerable breed and individual variations in the shape of the skull (17), making it more complex to measure or estimate bite force than in humans. Moreover, it is difficult to measure bite force while dogs and cats are awake. To overcome this limitation, several measuring methods have been developed in veterinary medicine. This mini-review covers the various measurement methods used in dogs and cats (Table 1) and elaborates on their advantages and limitations, as well as factors affecting bite force, and addresses the possible application of these methods in other fields.

**Table 1:** Studies on the bite force measurement/estimation in dogs and cats.

Animal	Measured/estimated location	Bite force (Newton, N)	Measurement/estimation method
Dog	Not specified	13–1,394	Measured by chewing transducer rolled with the rawhide (22)
	Canine teeth Molar teeth	147–926 574–3,417	Maximum bite force measurement by electronic stimulations (26)
	Canine teeth	300* 340* 571* 588*	Bite force estimation using equations of Kiltie (27) Thomason (28) Kiltie (26) (adjusted) Thomason (26) (adjusted)
	Molar teeth	755* 849* 1,949* 2,036*	Kiltie (27) Thomason (28) Kiltie (26) (adjusted) Thomason (26) (adjusted)
	Canine teeth Carnassial teeth	351.5* 549.8*	Bite force estimation using Thomason’s equation (29)
	Canine teeth Carnassial teeth	231.99–511.80^a^ 620.33–1,091.1^b^	Bite force estimation using finite element analysis (35)
Cat	Canine teeth Carnassial teeth	73.3* 118.1*	Maximal bite force estimation using Thomason’s equation (29)

*Values are the average of measured/estimated bite force.

*^a,b^Various estimated bite forces according to the gape angles between 5 and 65°*.

## Measurement of Bite Forces in Dogs and Cats

In humans, bite forces are typically measured using strain gauges, pressure-sensitive films, and piezoelectric sensors (18). In addition, the maximum voluntary bite force measured is used to diagnose TMJ disorders, mandibular fractures, and malocclusions (19–21). However, these methods are limited in that they can only measure vertical bite force. Because humans’ jaws move vertically and laterally, there is a need to measure bite force three-dimensionally. By contrast, jaws move mainly vertically and rarely laterally in dogs and cats; thus, there is little need to measure bite force in the lateral direction. No research has been done on the lateral forces. Nonetheless, it is challenging to measure bite forces in dogs and cats, as it is difficult or impossible to install a transducer in the mouth or force the animals to chew it deliberately and to specify the working or chewing side. Methods for measuring bite forces in dogs and cats are divided into two major types. First, bite force can be measured in the awake state or under anesthesia *in vivo*, and second, the bite force can be estimated using the calculation from a lever model or finite element analysis (FEA) model based on *in vitro* measurements.

### *In Vivo* Measurements of Bite Forces in Dogs and Cats

Lindner et al. measured bite forces in various dog sizes, without anesthesia, by chewing on a transducer (22) that could measure pressures when the dog bit it. The transducer had a 42-cm long, 2.5-cm diameter hollow steel rod, with a strain gauge on it, covered by a strip of steel for protection. The transducer was covered with a rubber tubing and overlaid with a beef-flavored rawhide chew for taste appeal. The test was performed in 22 dogs, weighing between 7 and 55 kg, and bite force was found to range from 13 to 1,394 Newtons (N), with a mean value of 256 N. Bite force varied widely in the dogs, increased slightly with body weight but was independent of the head configuration and jaw adductor muscle mass. The critical factor in bite force was the dog’s eagerness to chew the sensor wrapped in the chew, thus, chewing enthusiasm, personality, breed, and training are factors that can affect bite force (22). Due to the wide range of the reported bite forces and the high variability, a representative value is difficult to determine.

Other studies used a prosthetic implant transducer in three dogs (23, 24). In these studies, a force transducer, involving a titanium implant containing strain gauges, was implanted into the mandible 3 months after extraction of the mandibular third and fourth premolar teeth. Bite force was measured and recorded when the dog chewed on bones or dry dog food, over a period of several days. The measured bite force values reached 150 N when the dogs were chewing bones and 70 N when chewing dry dog food (24). However, these values cannot be considered representative of chewing bite force, because the location of the transducer was at the mandibular premolar teeth, rather than at an occlusal site, such as the first mandibular molar tooth and the maxillary fourth premolar tooth (i.e., carnassial teeth). These studies provided an experimental model for dental implant experiments and showed that several variables could affect bite force, including the location of the implant, occlusal conditions at the implant site, and the properties of the implant (23, 24).

Other studies have measured the maximal bite forces of dogs under general anesthesia by electrical stimulation of the jaw adductor muscles (25, 26). Strom and Holm (25) measured the bite forces by implanting silver electrodes into the masseter muscles in dogs. Muscle contraction was produced by intermittent stimulation of 2 ms, at a frequency of 20 Hz and gradually increasing voltage (up to 100 V). Muscle contraction stimulation was conducted for 5 min followed by a resting period of 10 min and was repeated four times over a total of 60 min. The bite force was determined to be 550 ± 35 N (mean ± SD) at the first stimulation; however, it gradually decreased to 100 ± 8 N at the fourth stimulation. Ellis et al. (26) also measured the bite force using electrical stimulation in 20 dogs weighing 5−40 kg. Under general anesthesia, four needle electrodes were inserted bilaterally into the masseter and temporal muscles. A force transducer was positioned between the maxillary fourth premolar teeth and the first molar teeth and the mandibular first and secondary molar teeth. Three electrical stimuli of 500 ms duration, at 60 Hz and 60 V amplitude each, were used to produce contraction with a period of 10-s rest in between. After that, the force transducer was repositioned to the canine teeth, and three more pulses were applied. The measured value ranged from 147 ± 6.9 to 926 ± 8.1 N on the canine teeth, and from 574 ± 83.2 to 3,417 ± 43.1 N on the carnassial teeth (26). Bite force significantly increased with body weight. These two studies were thus able to measure bite forces independent of volition, but the measure is of maximum bite force applied when all jaw adductor muscles were highly stimulated simultaneously, which likely rarely occurs intentionally in the awake dog (25, 26). Moreover, the force produced is dependent on the position of the electrodes in the muscles and the stimulation protocol, so it may not reflect a physiologic maximum voluntary contraction of a dog.

### *In Vitro* Measurements of the Bite Force

Several methods for *in vitro* measurement of bite force have been developed since the 1980s.

Kiltie (27) calculated the maximum bite force in sympatric species of neotropical cats, and Thomason (28) used the skulls of dogs and cats. These two authors used a “dry skull” to estimate the maximum bite force, using a lever model. They calculated the size of the jaw adductor muscles by the two-dimensional method; however, there were some differences between their methods. Kiltie used the lateral view of the skull to reflect the size of the temporal and masseter muscles, while Thomason used the dorsocaudal view of the skull for determining temporal muscle size and the ventral view of the skull for determining masseter muscle size (27, 28). Moreover, Kiltie’s study only considered unilateral bite force, while Thomason considered bilateral bite force. Furthermore, the length of the moment arm for each muscle differed, but the out-lever to the bite location was similar in these two studies (27, 28).

Ellis compared the bite force obtained using the equations derived from the studies by Kiltie (27) and Thomason (28) with results from *in vivo* measurements in dogs under anesthesia (26). After *in vivo* measurements, the dogs were euthanized, and then the bite force was obtained from two model equations using the skull of the same dog. It was demonstrated that the bite force obtained using the two model equations was lower than those obtained from *in vivo* measurements, likely due to the use of two-dimensional measurements (26) and other oversimplified assumptions. Therefore, the equations were adjusted using two methods and were further more accurately adjusted using iterative regression and including additional measurements but which were deemed only applicable to the set of dogs measured in the study (26).

Other studies have estimated the bite force from the skulls of carnivores in museum specimens (29–31). These studies were based on Thomason’s method; however, to compare the bite force across species with different body sizes, Wroe et al. developed the bite force quotient which resulted from a regression of calculated bite force with body weight (29, 31).

These studies have the advantage that these equations can be used to estimate the approximate bite force simply for comparison between species and can help to study the allometry in extinct species through their skulls. However, the calculated values are lower than the actual measurement values because it reflects only part of the muscle, two-dimensionally.

Finite element analysis is a numerical tool that can help to solve complex problems in vertebrate biomechanics (32). It allows elucidation of bite force from diverse vertebral skeletal tissues by building, loading, and validating the models using a computer system (32, 33). Several studies have used FEA for calculating bite force in canids and felids (34–36). Bourke et al. made a brick finite element model of a *Canis lupis dingo* skull using computerized serial tomography data based on the study by Wroe et al (36). They used FEA to calculate the bite force on canine and molar teeth according to the degree of gape and found that as the gape angle increased, the bite forces on the canine and molar teeth tended to decrease. The bite force was calculated to be in a range of 220–560 N for canine teeth and 310–1,100 N for the carnassial teeth (35). Therrien et al. (37) also estimated bite forces in five canids and felids; however, they stressed the biomechanics of mandibles more than jaw adductor muscles. The skulls were scanned with computed tomography (CT) and they focused on the mandibular body and the cortical bone within it. After calculation of bite forces with FEA, they found that these models could calculate more accurate bite forces than other models focusing on jaw adductor muscles (37).

## Factors Affecting Bite Force

In humans, bite force is an essential indicator of masticatory functional performance, and it is related to factors such as craniofacial morphology, periodontal support of teeth, dental status, malocclusion, TMJ dysfunction, age, and sex (38, 39). In dogs and cats, several factors affect the bite force, as seen in humans. First, the size and shape of the skulls and the body size depend on the breed, particularly in dogs (40). Ellis et al. calculated the bite force using the skulls of dogs, with adjustment of the equations used in a previous study (26). They sorted the skulls into small, medium, and large skulls by size and into brachy-, mesati-, and dolichocephalic skull shape. To distinguish the skull size, shape, and bite force, they measured the skull length, maximal skull width, and the length from rostral-most point of the skull to the caudal edge of the last maxillary molar tooth in skulls and compared the estimated bite forces using the adjusted equations (40). They found that in dogs, the bite force was closely related to the size of the skull and thus body weight. However, they also stated that obesity should not be included when determining body size; thus, the skull rather than body weight should be considered as the indicator (40). Bite force was increased as the skulls changed from dolichocephalic to brachycephalic shapes because the out-lever arm of the mandible in dolichocephalic skulls is longer than in brachycephalic skulls. However, the shape of the skull was not the significant factor determining bite force in small dogs (40). Because the brain case is relatively large regarding the facial structures in small brachycephalic dogs, there is a relatively small space for the masseter muscle and therefore a decreased bite force. In larger brachycephalic dogs, the brain case size did not influence the bite force (40).

Pain associated with oral diseases, such as periodontal disease, stomatitis, or TMJ disorders in dogs and cats can be a crucial factor determining bite force. The most common TMJ disorder is osteoarthritis (OA) in dogs and fracture in cats (41). TMJ OA is the second most common TMJ disorder in cats (41). Other TMJ disorders such as fracture, luxation, or neoplasia can result in pain when opening or closing the mouth and can decrease the range of motion in the joint (41, 42). Moreover, pain expressed in the TMJ may be related to masticatory muscles, as according to Hilton’s law, nerves that innervate the TMJ also innervate the masticatory muscles that move the joint to protect the TMJ from further damages (43, 44). According to the study by Goiato et al., there was a significant increase of the bite force 30 days after treatment in human patients with pain in the TMJ and masticatory muscles; thus, pain can affect bite force (45).

If dogs and cats have moderate to severe periodontal diseases, pain or discomfort and reduced periodontal support can occur concurrently in the oral cavity. The loading force during mastication is generated by the masticatory muscles, and mechanoreceptors on the periodontal ligament are involved in mastication (46). If a stimulus is applied, the receptors respond to a force applied to the crown of the tooth. However, if periodontal support is insufficient, it may result in a reduced control of bite force (46). In humans, studies have yielded contrasting results. According to the study by Alkan et al. (47), there were significantly reduced biting abilities in patients with chronic periodontitis, as compared to people with healthy periodontal tissues. By contrast, Kleinfelder and Ludwigt (48) have reported that a decreased periodontal attachment did not influence bite force in the natural dentition. Thus, we can assume that this periodontal status can be a factor influencing bite force; however, it can be controversial.

Masticatory muscle myositis (MMM) is an idiopathic and autoimmune disease in dogs and is one of the focal inflammatory myopathies (49, 50). This disease affects temporal, masseter, and medial and lateral pterygoid muscles, which have type 2 M fibers that contain a unique myosin, which differs from that of limb muscles (51). In dogs with MMM, autoantibodies specifically target 2 M fibers, leading to necrosis, phagocytosis, and fibrosis of affected muscles by infiltrating inflammatory cells (52). Dogs are presented with muscle swelling, pain, fever, and a decreased activity in the acute phase. In the chronic phase, clinical signs such as marked masticatory muscle atrophy and an inability to open the mouth are present (53). This disease can affect biting itself, if it is not treated adequately in the acute phase; as in the chronic phase, severe muscle atrophy occurs due to progressive fibrosis of the muscle fibers (50) and further reduces bite force.

## Application of Bite Force Measurements in Other Fields

Bite force has been used as an indicator in various fields in human and veterinary medicine. In humans, the bite force is used to evaluate the therapeutic effects of prosthetic devices, to suggest standards for the biomechanics of prosthetic devices, and is also used in maxillofacial surgery and dentistry (54, 55). In veterinary medicine, it can be used to evaluate restorative materials, develop fracture fixation implants, such as plates and screws, or to assess various foods, chewing toys, and dental treats for dogs and cats (22). Moreover, bite force knowledge has been used in evolutionary biology. Bite force can be estimated or measured from the dry skulls of extinct animals; thus, it enables the prediction of predatory behaviors or adaptation to feeding ecology in carnivores (29–31, 34).

## Conclusion

Bite force has been used as an important indicator for evaluating the masticatory system in dogs and cats as well as in humans. However, unlike humans, it is difficult to measure bite force in dogs and cats because chewing action cannot be directed. Thus, several measurement methods have been developed for use in the veterinary field. Bite force measurements in living dogs are challenging, but they allow the measurement of actual (i.e., real-life) bite forces. Bite force is more easily measured using dry skull measurements. However, these do not accurately mimic conscious bite force measurements, and measured bite forces from those measurements can be lower than the values from “live” measurements. Factors that can affect bite forces include the size and shape of the skull, body weight, and oral or TMJ pain. Masticatory muscle disease such as MMM can also cause mastication disability and altered bite force. Bite force values can be applied in various fields in animals as well as in humans. It can be used to evaluate dental materials, plates, and screws for fixation of craniomandibular fractures and in allometry studies that can estimate the bite force from extinct species.

## Author Contributions

SK: acquisition of data, analysis and interpretation of data, drafting of manuscript, critical revision of the manuscript for important intellectual content. BA: study concept and design, analysis and interpretation of data, critical revision of the manuscript for important intellectual content. TG: drafting of manuscript, acquisition of data, analysis and interpretation of data, critical revision of the manuscript for important intellectual content. FV: study concept and design, critical revision of the manuscript for important intellectual content, corresponding author.

## Conflict of Interest Statement

The authors declare that the research was conducted in the absence of any commercial or financial relationships that could be construed as a potential conflict of interest.
